# Draft Genome Sequence of *Roseovarius tolerans* EL-164, a Producer of *N*-Acylated Alanine Methyl Esters and *N*-Acylhomoserine Lactones

**DOI:** 10.1128/genomeA.01096-15

**Published:** 2015-09-24

**Authors:** Sonja Voget, Hilke Bruns, Irene Wagner-Döbler, Stefan Schulz, Rolf Daniel

**Affiliations:** a Genomic and Applied Microbiology and Göttingen Genomics Laboratory, Georg-August-University, Göttingen, Germany; b Institute of Organic Chemistry, Technical University Braunschweig, Germany; c Helmholtz Centre for Infection Research, Braunschweig, Germany

## Abstract

*Roseovarius tolerans* EL-164 is a member of the *Roseobacter* clade, a group of marine bacteria within the *Alphaproteobacteria*. It produces different *N*-acylhomoserine lactone (AHL) autoinducers as well as five AHL-related but functionally different compounds, the *N*-acylated alanine methyl esters. The size of the draft genome is 3,749,755 bp.

## GENOME ANNOUNCEMENT

The *Roseobacter* clade (family *Rhodobacteraceae*) is a group of marine bacteria within the *Alphaproteobacteria*. *Roseovarius* is one of the largest genera in the clade, which comprise four different lineages (1). *Roseovarius tolerans* was isolated from the hypersaline Ekho Lake (Antarctica) and was one of the first described species of the genus (2). *R. tolerans* EL-164 is one of the strains within this species lacking bacteriochlorophyll *a* production under standard laboratory conditions, although the *pufLM* genes of the photosynthesis gene cluster are present (3). *R. tolerans* EL-164 has been shown to produce *N*-acylhomoserine lactone (AHL) autoinducers with side chain lengths of 14 and 16 carbon atoms, and with and without an unsaturation (4). *R. tolerans* EL-164 was chosen for sequencing because of its ability to produce *N*-acylated alanine methyl esters (NAMEs). NAMEs are novel compounds, which are related to AHL autoinducers but show no quorum-sensing or quorum-quenching activity (4). Strain EL-164 was grown in Marine Broth (Difco 2216) at 20°C (3). Chromosomal DNA was isolated as described previously (3). Preparation of paired-end sequencing libraries with the Nextera XT library preparation kit and sequencing of the resulting libraries using the Genome Analyzer IIx were performed as recommended by the manufacturer (Illumina, San Diego, CA, USA). Sequencing resulted in 9,675,510 paired-end reads of 112 bp. *De novo* assembly with SPAdes version 2.5.0 (5) resulted in 121 contigs. The draft genome sequence of strain EL-164 exhibits a size of 3,749,755 bp and a G+C content of 63.9%. Protein-encoding genes were predicted and annotated with the Prokka annotation pipeline using Prodigal version 2.6 (6). The draft genome harbors 2 rRNA operons, 44 tRNA genes, and 3,685 predicted protein-encoding genes. In the genome the AHLs were assigned to autoinducer synthases and receptor proteins of the LuxI/LuxR-type (ROTO_12350/_12360 and ROTO_01950/_01960, respectively).

### **Nucleotide sequence accession number**s.

This whole genome shotgun project has been deposited in DDBJ/EMBL/GenBank under the accession LGVV00000000. The version described in this paper is the first version, LGVV01000000.
